# Evaluation of Circulating Adipokines and Abdominal Obesity as Predictors of Significant Myocardial Ischemia Using Gated Single-Photon Emission Computed Tomography

**DOI:** 10.1371/journal.pone.0097710

**Published:** 2014-05-19

**Authors:** Chi-Sheng Hung, Yen-Wen Wu, Jei-Yie Huang, Pei-Ying Hsu, Ming-Fong Chen

**Affiliations:** 1 Department of Internal Medicine (Cardiology Division), National Taiwan University Hospital and National Taiwan University College of Medicine, Taipei, Taiwan; 2 Department of Nuclear Medicine, National Taiwan University Hospital and National Taiwan University College of Medicine, Taipei, Taiwan; 3 Cardiology Division of Cardiovascular Medical Center and Department of Nuclear Medicine, Far Eastern Memorial Hospital, New Taipei City, Taiwan; 4 National Yang-Ming University School of Medicine, Taipei, Taiwan; 5 Department of Nuclear Medicine, National Taiwan University Hospital, Yun-Lin Branch, Douliou City, Taiwan; Thomas Jefferson University, United States of America

## Abstract

**Objective:**

Coronary artery disease (CAD) is associated with abdominal obesity and metabolic syndrome. Adipocytes secrete adipokines, including the newly discovered adipocyte fatty acid binding protein (A-FABP) and chemerin. Adipokines contribute to the pathogenesis of CAD. In patients with CAD, the presence of significant ischemia predicts adverse outcomes. It is unknown whether adipokines can be better predictors of the presence of significant myocardial ischemia than conventional risk factors. This study aimed to compare adipokines with clinical risk factors and abdominal obesity as predictive factors for significant myocardial ischemia.

**Methods:**

One hundred and ninety-six adults with suspected, but unproven, CAD were consecutively enrolled. The main measures were clinical and biochemical parameters and stress myocardial perfusion imaging with gated myocardial perfusion single-photon emission computed tomography (SPECT), with computed tomography (CT) attenuation correction. The abdominal visceral fat area was examined using a hybrid SPECT/CT scanner. Serum levels of high-sensitivity C-reactive protein (hs-CRP) and adipokines (adiponectin, A-FABP, and chemerin) were evaluated.

**Results:**

A-FABP levels correlated significantly with adiponectin, hs-CRP, body mass index, waist circumference, and visceral fat area. A-FABP was significantly associated with metabolic syndrome (OR 3.2, 95% CI 1.6–6.4, *p* = 0.001), significant myocardial ischemia (OR 1.9, 95% CI 1.0–3.4, *p* = 0.05), and stress lung-to-heart ratio (β = 0.03, *p* = 0.03) on SPECT. Chemerin was significantly associated with serum triglyceride levels but not with metabolic syndrome, significant ischemia, or stress lung-to-heart ratio on SPECT. A-FABP was better at detecting significant inducible ischemia than other biomarkers, although this was a modest improvement (area under ROC curve 0.579, 95% CI 0.46–0.69).

**Conclusions:**

Serum A-FABP concentrations correlate significantly with visceral fat area, metabolic syndrome, and predicted significant myocardial ischemia on SPECT. This may help to more accurately assess CAD risk, especially in patients with metabolic syndrome.

## Introduction

Obesity, a state of excessive adipose tissue, has long been known to be a risk factor for the development of cardiovascular disease [1], [2]. Adipose tissue has been traditionally considered a fat-storage organ, but is now known to have an active role in systemic metabolism through the active secretion of adipokines. These adipokines can target distant organs and have major effects on body weight, energy storage, insulin sensitivity, glucose regulation, and the inflammatory response. Evidence also supports the notion that the adipose tissue of different body compartments has different adipokine secretion profiles [3]. Among the fat storage compartments in the body, visceral fat has been found to be an important source of proinflammatory adipokines [4], [5]. Visceral fat is associated with an increased risk of atherosclerosis compared to subcutaneous fat [6], [7]. According to these findings, the dysregulation of adipokines has been proposed as a potential link between obesity and cardiovascular disease [8]. Therefore, it is important to clarify the roles of different adipokines in the mechanism of metabolic disorders.

Two novel adipokines, adipocyte fatty acid binding protein (A-FABP) and chemerin, have been recently identified to be associated with adipose tissue, metabolic syndrome, and cardiovascular disease. A-FABP is reported to be associated with coronary artery disease (CAD) [9]–[11] and as a predictor for increased risk of cardiovascular death during long-term follow-up (hazard ratio, 1.75; 95% CI, 1.17–2.62, *p* = 0.007) [12]. Chemerin is involved in multiple aspects of various biological processes, including immunity, obesity, inflammation, and insulin resistance [13]. Serum chemerin has also been reported to be associated with the severity of coronary atherosclerosis [14]. However, the clinical utility of these two adipokines in the diagnosis or management of CAD has not yet been thoroughly explored.

In patients with CAD, the severity of inducible myocardial ischemia is an important predictor of adverse outcomes [15]. Myocardial perfusion imaging performed with thallium-201 (^201^Tl) reveals the severity of myocardial ischemia and helps identify patients who have increased risk of cardiac death or non-fatal myocardial infarction [16]. Patients with an ischemic area that is greater than 10% of the left ventricular myocardium, as detected by myocardial perfusion imaging, have better outcomes with revascularization than with medical therapy only [17]. Despite the fact that clinical scores (e.g., Framingham risk score) are useful to estimate the risk for CAD, no serum biomarker has been reported to reliably identify patients with significant myocardial ischemia.

In view of the emerging role of A-FABP and chemerin in patients with CAD, we designed this cross-sectional study to explore the presence of associations of these two adipokines with the amount of abdominal visceral fat, and with the severity of myocardial ischemia. We hypothesized that serum A-FABP and chemerin concentrations indeed follow these correlations, and that they would serve as useful biomarkers for the identification of patients with significant myocardial ischemia who may benefit from revascularization.

## Methods

### Ethical statement

The study was approved by the Institutional Review Board of the National Taiwan University Hospital and complies with the Helsinki Declaration. Written informed consent was obtained from each patient before enrollment.

### Study design

Between January 2008 and December 2011, we enrolled 196 patients with typical symptoms indicative of ischemic heart disease at the Cardiovascular Center of National Taiwan University Hospital, Yun-Lin Branch, Taiwan. Subjects with the following conditions were excluded from this study: unstable vital signs, acute coronary syndrome, a history of myocardial infarction (MI), severe heart failure (NYHA functional class 4), severe asthma or chronic obstructive pulmonary disease, high-degree atrial-ventricular conduction block without a pacemaker, or pregnancy. In addition to clinical and biochemical parameters, all subjects received anthropometric evaluation and underwent nuclear myocardial perfusion imaging. Waist and hip circumferences were measured to the nearest 0.1 cm. Blood pressure was recorded using a mercury sphygmomanometer to the nearest 2 mmHg with the arm supported at heart level after sitting quietly for 10 minutes. Plasma glucose and the fasting serum total cholesterol, triglycerides, high-density lipoprotein (HDL) cholesterol, and low-density lipoprotein (LDL) cholesterol were measured with an automated analyzer (Toshiba TBA 200 FR, Toshiba Medical Systems Co., Ltd., Tokyo, Japan). Plasma glycosylated hemoglobin (HbA1c) was measured using another automated analyzer (HLC-723 G7 HPLC Systems, Tosoh Corporation, Tokyo, Japan). Diabetes was diagnosed according to the 2010 American Diabetes Association (ADA) recommendations (HbA1c≥6.5%, fasting plasma glucose ≥126 mg/dL, or 2-hour plasma glucose after standard 75 g oral glucose tolerance test ≥200 mg/dL) [18]. Metabolic syndrome was defined according to the ATP III study panel, with modification of the waist circumference criteria to match Asian ethnicity, as meeting three or more of the following criteria: 1) high blood pressure: systolic and/or diastolic blood pressure ≥130/85 mmHg or receiving blood pressure-lowering medications, 2) hyperglycemia: fasting plasma glucose ≥100 mg/dL (5.6 mmol/L) or receiving glucose lowering medications, 3) hypertriglyceridemia: fasting plasma triglyceride ≥150 mg/dL (1.69 mmol/L), 4) HDL cholesterol <40 mg/dL in men and <50 mg/dL in women, and 5) waist circumference ≥90 cm in men and ≥80 cm in women [19].

Subjects' samples were collected in potassium-EDTA tubes after overnight fasting and stored at −80°C until assayed. Serum biomarkers were analyzed by commercially available enzyme-linked immunosorbent assay (ELISA) kits as follows: A-FABP (BioVendor Laboratory Medicine, Inc., Brno, Czech Republic), chemerin (Millipore, Corporation, Billerica, MA, USA), adiponectin (B-Bridge, Tokyo, Japan), and hs-CRP (Millipore, Corporation, Billerica, MA, USA). Inter-assay and intra-assay coefficients of variation of all assays were less than 5% and all laboratory work was undertaken by researchers that were blinded to the patients' clinical details.

### Nuclear myocardial perfusion imaging

A dipyridamole stress ^201^Tl single-photon emission tomography (SPECT) was performed within one month after clinical evaluation. Dipyridamole (0.14 mg/kg/min) was intravenously infused over 4 minutes to induce coronary hyperemia. Three minutes after the completion of the infusion, 2.5–3 mCi ^201^Tl was injected. ECG-gated SPECT (8 frames per cardiac cycle) was acquired 5 minutes and 3–4 hours after ^201^Tl injection in all patients on a hybrid dual-head SPECT/CT scanner (Symbia T2, Siemens Medical Solutions, Inc. Hoffman Estates, IL, USA) equipped with 2-slice CT. Low-dose CT scans of the chest region were routinely acquired after stress SPECT for attenuation correction [20].

In addition to measuring LV functional parameters and stress lung-to-heart ratio [20], the myocardium was divided into 17 segments and each segment was semi-quantitatively visually scored using a 5-point scoring system (0 =  normal, 1 =  mildly abnormal, 2 =  moderately abnormal, 3 =  severely abnormal, 4 =  absence of tracer uptake). The summed scores at rest (SRS) and after stress (SSS) were obtained by adding the individual segment score at rest and after stress, respectively. The summed difference score (SDS) was calculated by subtracting the SRS from the SSS. The segments with reverse distribution were excluded from the calculation of SDS. The extent of ischemia was expressed as the percentage of ischemic myocardium (SDS/68×100). The extent of ischemia was then classified into 3 levels by the percentage of ischemic myocardium, with 0% being no ischemia, 1%–9% being mild ischemia, and ≥10% being significant ischemia, which evidence suggests is the threshold of ischemia where revascularization shows greater benefit than medical treatment alone [17].

### Measurement of abdominal adipose tissue areas

Abdominal adiposity was assessed using the same SPECT/CT scanner throughout the study, with the patient lying in a supine position immediately after stress myocardial perfusion imaging. Twenty-five contiguous 5-mm thick slices (120 kVp, 400 mA, gantry rotation time 500 ms, table feed 3∶1) were acquired, covering 125 mm above the level of S1. The raw data were reconstructed using a 55-cm field-of-view. Subcutaneous fat was defined as the extra-peritoneal fat between the skin and muscle, with attenuation ranging from −195 to −45 Hounsfield units (HU) and a window center of −120 HU to identify pixels containing adipose tissue. In order to separate visceral from subcutaneous fat, the abdominal muscular wall separating the two compartments was manually traced. The visceral adipose tissue (VAT) area and subcutaneous adipose tissue (SAT) area were determined by automatic planimetry at the umbilical level. The intra- and inter-reader reproducibility was high for the SAT and VAT measurements in our laboratory (inter-reader and intra-reader comparisons, all *r*≥0.98, *p*<0.0001) [21].

### Statistical analysis

All variables are expressed as the mean ± standard deviation (SD) in SI units. Pearson's correlation tests were used to assess the correlations between the metabolic parameters and A-FABP or chemerin after adjusting for age. Diagnostic criteria of significant myocardial ischemia using clinical parameters, serum biomarkers, and abdominal fat areas were developed based on receiver operating characteristic (ROC) analysis. Logistic regression models were used to evaluate associations between significant ischemia and the presence of metabolic syndrome and biomarkers levels. Linear regression analyses were performed to evaluate associations between stress lung-to-heart ratio and biomarker levels.

## Results

Overall, 89 women and 97 men were enrolled. The demographic and clinical characteristics of the study subjects are shown in Table 1. The mean age was 63.6 (±10.7) years. Among all subjects, 48% had hypertension, 31% had diabetes, 21% had dyslipidemia, and 67% had metabolic syndrome. Compared to women, men had significantly lower SAT areas (80.2±35.1 cm^2^ versus 121.4±45.6 cm^2^, *p*<0.001), but tended to have higher VAT areas (74.9±31.6 cm^2^ versus 67.6±26.9 cm^2^, *p* = 0.08). The average resting LV ejection fraction was 62.3% (57.6% for men and 67.1% for women, *p*<0.001), a gender difference that was also observed in our previous report [20], [22]. The mean SSS was 6.7 (7.9 for men and 5.4 for women) and the mean SDS was 3.2 (3.2 for men and 3.1 for women), with no differences between men and women (*p*≥0.05). The numbers of patients with no ischemia, mild ischemia, and significant ischemia were 47 (25.3%), 114 (61.3%), and 25 (13.4%), respectively.

**Table 1:** Baseline characteristics, abdominal fat areas, and myocardial perfusion imaging.

	Men (n = 97)	Women (n = 89)	*p*
Age, year	62.5 (11.7)	64.8 (9.4)	0.13
Systolic blood pressure, mmHg	132.3 (16.8)	139 (20.6)	0.02
Diastolic blood pressure, mmHg	78.3 (11.5)	78.9 (14.2)	0.76
Waist circumference, cm	95.3 (8.5)	90.7 (10.5)	0.001
Body mass index, kg/m^2^	26.3 (3.6)	27.3 (4.2)	0.08
Comorbidity, n (%)			
Hypertension	38 (39.1%)	52 (58.4%)	0.009
Diabetes	28 (28.8%)	30 (33.7%)	0.48
Dyslipidemia	17 (17.5%)	22 (24.7%)	0.23
Metabolic syndrome	61 (62.9%)	64 (71.9%)	0.19
Smoking	24 (24.7%)	2 (2.2%)	<0.001
History of percutaneous coronary intervention	16 (16.5%)	5 (5.6%)	0.02
Medication, n (%)			
Calcium channel blocker	21 (21.6%)	26 (29.2%)	0.24
Angiotensin-converting enzyme inhibitor	5 (5.2%)	4 (4.5%)	0.83
Angiotensin II receptor blocker	14 (14.4%)	16 (18%)	0.51
Beta-blocker	22 (22.7%)	14 (15.7%)	0.23
Alpha-blocker	5 (5.2%)	1 (1.1%)	0.12
Aspirin	45 (46.4%)	38 (42.7%)	0.61
Statin	17 (17.5%)	14 (15.7%)	0.74
Fibrate	4 (4.1%)	0 (0%)	0.12
Sulfonylurea	11 (11.3%)	8 (9%)	0.6
Metformin	15 (15.5%)	13 (14.6%)	0.87
Thiazolidinedione	4 (4.1%)	2 (2.2%)	0.47
Insulin	2 (2.1%)	1 (1.1%)	1
Serum biomarkers, mean (SD)			
Creatinine, mg/dL	1.34 (1)	1.07 (0.4)	0.02
Total cholesterol, mg/dL	190.8 (44)	207 (94.4)	0.2
LDL-C, mg/dL	113.6 (42.1)	114.9 (48.2)	0.9
HDL-C, mg/dL	41.3 (7.4)	51.7 (24.1)	0.003
Triglyceride, mg/dL	158.1 (90.7)	167.6 (242)	0.76
Fasting glucose, mg/dL	117.3 (41.4)	113.8 (50.9)	0.65
HbA1c, %	6.9 (1.6)	6.9 (1.4)	0.94
hs-CRP, mg/L	4.8 (9.9)	4.4 (17.3)	0.75
A-FABP, ng/mL	22.7 (14.4)	41.9 (35.4)	<0.001
Adiponectin, µg/mL	6.6 (3.9)	10.8 (7.9)	<0.001
Chemerin, ng/mL	52.7 (14.4)	56.1 (16.8)	0.16
Abdominal computed tomography for fat area, mean (SD)			
Visceral adipose tissue area, cm^2^	74.9 (31.6)	67.6 (26.9)	0.08
Subcutaneous adipose tissue area, cm^2^	80.2 (35.1)	121.4 (45.6)	<0.001
Myocardial perfusion image, mean (SD)			
LVEF, %	57.6 (11.9)	67.1 (13.6)	
SSS	7.9 (10.4)	5.4 (5.8)	0.08
SDS	3.2 (4.3)	3.1 (3.9)	0.77
Stress lung-to-heart ratio	0.37 (0.07)	0.36 (0.07)	0.42

SD, standard deviation; LDL-C, low-density lipoprotein cholesterol; HDL-C, high-density lipoprotein cholesterol; HbA1c, hemoglobin A1c; hs-CRP, high-sensitivity C-reactive protein; A-FABP, adipocyte fatty acid-binding protein; LVEF, left ventricular ejection fraction; SSS, summed stress score; SDS, summed difference score.

Age-adjusted correlations of A-FABP or chemerin with metabolic parameters, other adipokines, and abdominal fat areas are listed in Table 2. A-FABP correlated significantly with adiponectin, hs-CRP, body mass index (BMI), waist circumference, and abdominal fat areas in all participants, but did not correlate with lipid profiles. In addition, the correlation of A-FABP with adiponectin and hs-CRP was gender-dependent. No correlations were found between chemerin and hs-CRP, A-FABP, or adiponectin. Chemerin only correlated with triglyceride levels in males.

**Table 2:** Age-adjusted Pearson's correlation between A-FABP and chemerin with metabolic parameters.

	Overall		Men		Women	
	A-FABP	Chemerin	A-FABP	Chemerin	A-FABP	Chemerin
Adiponectin	0.301*	−0.05	0.0690	−0.1317	0.2526*	−0.0631
hs-CRP	0.197*	0.05	0.3486*	−0.0685	0.1151	0.1960
Total cholesterol	−0.103	0.07	−0.1060	0.2959*	−0.1674	−0.0245
Triglyceride	0.164	0.238*	0.2647	0.3313*	0.1254	0.1948
Body mass index	0.445*	0.065	0.3857*	0.0817	0.4608*	0.0234
Waist circumference	0.319*	0.03	0.4522*	0.0723	0.4787*	0.0570
Systolic blood pressure	0.053	0.112	−0.0009	−0.0279	−0.0188	0.2102
Diastolic blood pressure	−0.023	0.07	−0.0493	0.1262	−0.0101	0.0382
Visceral adipose tissue area	0.285*	0.14	0.4534*	0.2212*	0.3349*	0.0816
Subcutaneous adipose tissue area	0.481*	0.009	0.4292*	0.1436	0.2927*	−0.2190

*p<0.05; hs-CRP, high-sensitivity C-reactive protein; A-FABP, adipocyte fatty acid-binding protein.

Table 3 displays the associations between serum A-FABP or chemerin and the presence of metabolic syndrome. In the crude analysis (Model 0), the odds ratios (OR) for metabolic syndrome was 3.2 (1.6–6.4, *p* = 0.001) with A-FABP and 2.7 (0.8–8.7, *p* = 0.1) with chemerin. The BMI and VAT areas were also significantly associated with metabolic syndrome (excluding factors directly included in the criteria of metabolic syndrome). Further analyses were performed in different models: Model 1 was adjusted for age, Model 2 was adjusted for age and BMI, and Model 3 was adjusted for age and VAT area. The results revealed significant associations between A-FABP and metabolic syndrome in total participants and in male participants. The associations between chemerin and metabolic syndrome were not significant in these analyses.

**Table 3:** Predictive ability of A-FABP and chemerin for metabolic syndrome.

	Overall OR (95% CI)	*p*	Men	*p*	Women	*p*
Model 0						
A-FABP	3.2 (1.6–6.4)	0.001	6.9 (1.8–25.1)	0.004	2.3 (0.9–5.8)	0.09
Chemerin	2.7 (0.8–8.7)	0.1	2.6 (0.6–12.6)	0.2	2.4 (0.4–14.7)	0.34
Model 1						
A-FABP	3.3 (1.6–6.7)	0.001	8.7 (2.2–33.7)	0.002	1.9 (0.7–5.1)	0.2
Chemerin	2.6 (0.8–8.5)	0.12	3.4 (0.7–17.3)	0.1	2.1 (0.3–13.5)	0.4
Model 2						
A-FABP	2.3 (1.1–5.0)	0.03	5.5 (1.4–22.7)	0.02	1.3 (0.4–3.9)	0.6
Chemerin	2.4 (0.7–8.5)	0.17	2.85 (0.5–15.6)	0.3	2.1 (0.3–15.7)	0.5
Model 3						
A-FABP	2.3 (1.1–5.0)	0.03	5.2 (1.2–22)	0.02	0.9 (0.3–3.0)	0.9
Chemerin	1.7 (0.5–6.0)	0.41	2.0 (0.4–11.4)	0.4	1.3 (0.2–9.5)	0.3

Model 0: no adjustment.

Model 1: adjusted for age.

Model 2: adjusted for age, body mass index.

Model 3: adjusted for age, visceral adipose tissue area.

A-FABP, adipocyte fatty acid-binding protein.

Table 4 shows the associations between A-FABP or chemerin and the presence of significant ischemia. In the crude analysis (Model 0), the OR for significant ischemia was 2.0 (1.0–3.9, *p* = 0.05) with A-FABP and 3.2 (0.6–17.1, *p* = 0.17) with chemerin. The VAT area, SAT area, serum hs-CRP, and adiponectin concentration were not significantly associated with the presence of significant ischemia. Other parameters that correlated with the presence of significant ischemia included systolic blood pressure (SBP), diastolic blood pressure, and a history of CAD. We adjusted these factors in Models 1–3: Model 1 was adjusted for age, Model 2 was adjusted for age and SBP, and Model 3 was adjusted for age, SBP, and a history of CAD. These adjustments for the covariates revealed significant associations between A-FABP and the presence of significant ischemia in total participants and in female participants. Chemerin, however, showed a significant association with the presence of significant ischemia only in Model 2 for total participants. The diagnostic ability of A-FABP to identify patients with significant ischemia was modest (area under the ROC curve 0.57, 95% CI 0.43–0.71), and the optimal cutoff point determined by the Youden method was 45.6 ng/ml (sensitivity 43.5%, specificity 76.8%). We further evaluated the association between the stress lung-to-heart ratio on SPECT and clinical parameters and biomarkers. In the univariate analyses, waist circumference, A-FABP concentration, and hs-CRP were significantly associated with stress lung-to-heart ratio. To investigate these associations further, multivariate linear regression analyses were performed using stress lung-to-heart ratio as the dependent variable and variables with a *p* value of less than 0.1 in the univariate regression analysis as independent variables. After adjusting for possible confounding factors, A-FABP concentration was still significantly associated with stress lung-to-heart ratio (Table 5).

**Table 4:** Predictive ability of A-FABP and chemerin for significant ischemia.

	Overall OR (95% CI)	*p*	Men	*p*	Women	*p*
Model 0						
A-FABP	2.0 (1.0–3.9)	0.05	2.9 (0.8–9.8)	0.09	2.1 (0.8–5.5)	0.14
Chemerin	3.2 (0.6–17.1)	0.17	1.8 (0.2–16.6)	0.58	4.2 (0.4–40.8)	0.22
Model 1						
A-FABP	2.3 (1.1–4.8)	0.03	2.7 (0.7–9.6)	0.13	4.1 (1.3–13.7)	0.02
Chemerin	3.7 (0.7–20.2)	0.13	1.4 (0.2–14.1)	0.73	6.6 (0.6–78.7)	0.14
Model 2						
A-FABP	2.3 (1.1–4.8)	0.03	2.4 (0.7–8.3)	0.18	4.9 (1.2–18.7)	0.02
Chemerin	5.1 (1.1–24.7)	0.04	1.7 (0.2–16.1)	0.63	7.5 (0.6–102.6)	0.13
Model 3						
A-FABP	2.3 (1.1–4.8)	0.04	2.0 (0.6–7.4)	0.30	5.2 (1.3–20.5)	0.02
Chemerin	4.4 (0.8–24.4)	0.09	2.3 (0.2–24.4)	0.48	5.8 (0.6–60.1)	0.14

Model 0: no adjustment.

Model 1: adjusted for age.

Model 2: adjusted for age, systolic blood pressure.

Model 3: adjusted for age, systolic blood pressure, history of coronary artery disease.

A-FABP, adipocyte fatty acid-binding protein.

**Table 5:** Associations of clinical characteristics and biomarkers with increased stress lung-to-heart ratio during myocardial perfusion imaging.

	Univariate			Multivariate		
	Coef.	95% CI	*p*	Coef.	95% CI	*p*
Age	−0.0004	−0.001–0.0005	0.40	0.0002	−0.01–0.001	0.84
Sex	0.009	−0.0136–0.032	0.43	−0.128	−0.061–0.035	0.60
Waist circumference	0.0015	0.0003–0.0027	0.01	−0.001	−0.0035–0.0013	0.36
Visceral fat area	0.0002	−0.0001–0.0006	0.20			
Systolic blood pressure	0.0001	−0.0005–0.0006	0.82			
HbA1c	0.0081	−0.0006–0.0168	0.07	0.015	0.002–0.029	0.03
A-FABP*	0.022	0.0016–0.043	0.03	0.056	0.013–0.099	0.01
hs-CRP*	0.009	0.0003–0.017	0.04	0.002	−0.009–0.137	0.67
Chemerin*	−0.007	−0.048–0.034	0.73			
Adiponectin*	−0.0007	−0.22–0.21	0.95			

HbA1c, hemoglobin A1c; hs-CRP, high-sensitivity C-reactive protein; A-FABP, adipocyte fatty acid-binding protein.

## Discussion

We demonstrated significant associations of A-FABP with metabolic syndrome and with significant ischemia in patients with stable angina referred for myocardial perfusion imaging examination. The association was also gender-dependent; women showed a much stronger association with significant ischemia, while men show a stronger association with metabolic syndrome. The associations between A-FABP and SAT and VAT were significant after adjusting for age and gender. However, the association of chemerin with metabolic syndrome or significant ischemia was weak and not statistically significant. Our study is the first to measure serum adipokines, VAT, and myocardial ischemia simultaneously on a hybrid SPECT/CT protocol. Although VAT has been previously reported to correlate strongly with CT coronary atherosclerosis [23], we did not find a significant association between VAT and the presence of significant ischemia. Our data demonstrate a stronger correlation between myocardial ischemia and serum A-FABP compared with the correlation between myocardial ischemia and VAT; these findings support the notion that A-FABP is strongly linked to human coronary atherosclerosis. Although its associations were statistically significant, the predictive ability of A-FABP for significant ischemia was only modest.

A-FABP has been associated with atherosclerosis in animal and clinical studies. A-FABP mRNA and protein are expressed in the VAT of human biopsy specimens [24], and A-FABP protein can be released into the circulation by adipose tissue [25]. A-FABP4 is also implicated in the development of insulin resistance and atherosclerosis in animal studies. In A-FABP knockout mice with comorbid leptin deficiency, the animals became obese compared with those with control mice with leptin deficiency only. Insulin sensitivity, however, improved significantly in the A-FABP knockout mice despite their obesity [26]. In an A-FABP and ApoE double-knockout mouse model, the mean atherosclerotic lesion area in the aorta decreased by 66% in double-knockout mice compared with controls [27]. Recently, several clinical studies have demonstrated an association between A-FABP and coronary atherosclerosis. In a report by Jin *et al.*, the A-FABP concentration was higher in female CAD patients than in non-CAD subjects and was also independently associated with Gensini scores (a coronary artery stenosis score) [9]. Circulating A-FABP concentrations were significantly higher in patients with CAD, and correlated with coronary atherosclerosis index [10] and with the number of diseased coronary vessels [11]. In a multivariate logistic regression analysis, A-FABP concentration was an independent predictor for CAD in women even after adjusting for waist circumference, HbA1c, insulin resistance, LDL, glomerular filtration rate, and N-terminal pro-brain natriuretic peptide [10]. Our results complement these studies and show that A-FABP is not only associated with the presence of CAD but also significantly associated with the presence of clinically significant ischemia. The association remained significant after adjusting for age, blood pressure, and history of CAD. This association was significant in the total participant group and in women but not in men. Although the small patient number in the present study may be responsible for the differences in associations between women and men, a gender-dependent association was also observed in the results of Bao *et al*. [10]. It is well known that body fat distribution, abdominal adipose tissue in particular, differs between human males and females. Women have less visceral fat accumulation and a lower ratio of VAT to total body fat compared with men [28], [29]. Sexual dimorphism has also been reported for adipokines, including leptin [30], adiponectin [31], and chemerin [32]. These gender differences in adipokines may reflect gender differences in adipocyte function or sex hormone regulation [33]. Our results corroborate previous reports [10], suggesting that A-FABP may have a more prominent role in the pathogenesis of myocardial ischemia in females compared with males. In addition, A-FABP may be involved in atherosclerosis during the advanced stages, when a considerable amount of myocardium is at risk.

We tested the potential clinical use of A-FABP or chemerin in identifying patients with significant ischemia. Current guidelines utilize the presence of significant ischemia as one of the factors that determine if a patient with CAD needs to receive percutaneous coronary intervention [15]. Patients with larger areas of ischemic myocardium are at higher risk for future cardiovascular events, and thus benefit from revascularization. In patients with CAD, biomarkers, such as pro-atrial natriuretic peptides or brain natriuretic peptide (at baseline or during exercise), have been tested to identify the presence of significant ischemia with only partial success [34]–[36]. According to our results, the ability of A-FABP or chemerin to identify patients with significant ischemia was modest.

Our group has recently reported that serum A-FABP levels are associated with moderate to severe myocardial ischemia and are an independent predictor for heart failure in patients with known CAD [37]. The present study confirmed these results in an independent study population with preserved cardiac function and without a history of MI, extending the scope of A-FABP's role to an earlier CAD stage. Furthermore, compared with myocardial ischemia's association with VAT, measured in the present study by CT, its association with A-FABP was stronger and more consistent. Our findings indicate that A-FABP is not merely a marker of VAT or VAT-related cardio-metabolic risk factors. Preclinical studies have already shown that A-FABP has the ability to promote cholesterol accummulation, inhibit cholesterol efflux, and induce proinflammatory cytokines in macrophages [38]. Taken together, these results suggest that A-FABP may contribute directly to the pathogenesis of coronary atherosclerosis and clinically significant myocardial ischemia.

Heightened chemerin levels have been reported to associate with metabolic syndrome [39] and inflammation [40]. Nevertheless, associations between chemerin and myocardial ischemia are not consistent in the literature. Three studies in Chinese populations reported elevated serum chemerin levels to be associated with the presence of CAD [41]–[43]. However, in another study of 303 Caucasian patients with chest pain, Lehrke *et al*. found that serum chemerin levels correlated significantly with inflammatory markers, including hs-CRP, interleukin-6, and tumor necrosis factor-α. Despite this link with inflammation, chemerin did not associate with severity of myocardial ischemia after adjusting for established cardio-metabolic risk factors [44]. Additionally, in a Korean study of 131 patients with known CAD, serum chemerin levels were not independently associated with CAD severity [45]. Our results are in agreement with these last two studies. Possible explanations for these inconsistent data include the different assays used, the different ethnic groups studied, and the different stages of coronary atherosclerosis exhibited by the patients enrolled in the studies. Even in those studies showing significant associations, the chemerin levels between CAD and non-CAD groups overlapped markedly. Taken together, these data and our results suggest that using chemerin to identify patients with significant ischemia is unlikely to be effective.

The present study has several limitations. First, to limit radiation exposure to health individuals, we did not enroll healthy subjects to serve as a control group, hence the serum levels of adipokines in healthy controls were not available in our study. Second, this cross-sectional study design does not allow the inference of a causal relationship between A-FABP and clinically significant ischemia. Third, coronary angiography was not routinely performed in the present study. However, we used ECG-gated SPECT and low-dose CT for attenuation correction, which helped to improve diagnostic accuracy, especially in obese or female subjects [20], [22], [46]. Finally, the present study lacked long-term follow-up to compare patient survival and cardiac events and to further evaluate the biomarkers' prognostic value.

## Conclusions

The newly discovered adipokine A-FABP is significantly associated with visceral fat areas, metabolic syndrome, and the presence of significant myocardial ischemia. The association between myocardial ischemia and serum adipokines supports a strong link connecting adipokine imbalance with human coronary atherosclerosis and myocardial ischemia. Using serum A-FABP concentration may help to more accurately assess CAD risk, especially in patients with metabolic syndrome.
